# STM Images of Anionic Defects at CeO_2_(111)—A Theoretical Perspective

**DOI:** 10.3389/fchem.2019.00212

**Published:** 2019-06-06

**Authors:** Matthew J. Wolf, Christopher W. M. Castleton, Kersti Hermansson, Jolla Kullgren

**Affiliations:** ^1^Department of Chemistry–Ångström Laboratory, Uppsala University, Uppsala, Sweden; ^2^School of Science and Technology, Nottingham Trent University, Nottingham, United Kingdom; ^3^Division of Physics and Mathematics/Natural Science Didactics, Mälardalen University, Västerås, Sweden

**Keywords:** simulated STM images, cerium dioxide (CeO2), density functional theory, anionic defects, reducible oxide

## Abstract

We present a theoretically oriented analysis of the appearance and properties of plausible candidates for the anionic defects observed in scanning tunneling microscopy (STM) experiments on CeO_2_(111). The simulations are based on density functional theory (DFT) and cover oxygen vacancies, fluorine impurities and hydroxyl groups in the surface and sub-surface layers. In the surface layer, all three appear as missing spots in the oxygen sublattice in filled state simulated STM images, but they *are* distinguishable in empty state images, where surface oxygen vacancies and hydroxyls appear as, respectively, diffuse and sharp bright features at oxygen sites, while fluorine defects appear as triangles of darkened Ce ions. In the sub-surface layer, all three defects present more complex patterns, with different combinations of brightened oxygen ion triangles and/or darkened Ce ion triangles, so we provide image maps to support experimental identification. We also discuss other properties that could be used to distinguish the defects, namely their diffusion rates and distributions.

## 1. Introduction

The functional properties of technologically relevant materials can depend critically on the properties of point defects in the lattice structure, which may be present unavoidably as contaminants or introduced deliberately as dopants. For this reason, much research effort goes into identifying and characterizing such defects. In the bulk, typically one must rely on spectroscopic probes, which provide spatially averaged information, but at the surface one can make use of spatially resolving techniques such as scanning tunneling microscopy (STM) and atomic force microscopy (AFM). Of these latter two methods, STM remains more widely used, despite the fact that its applicability is limited to materials with sufficient conductivity, and atomic resolution STM imaging of surfaces is now fairly routine. However, the interpretation of the results of STM experiments is complicated by the fact that the tunneling current depends not only on the topography of the surface, but also on the local electronic structure. Substantial success has been achieved by combining experimental data with the results of electronic structure calculations, most commonly based on density functional theory (DFT) (see e.g., Setv́ın et al., 2017 for a particularly relevant review of such studies on oxide surfaces).

The focus of the present contribution is the identification of anionic defects at the (111) surface facet of CeO_2_ (ceria) observed using STM. The earliest such experimental studies were carried out on single crystals obtained commercially, and mainly with negative bias voltages, thereby imaging the filled electronic states (Nörenberg and Briggs, 1997, 1998, 1999; Nörenberg, 2002; Fukui et al., 2003; Namai et al., 2003; Esch et al., 2005). The predominant defects observed were dark “depressions” in this sublattice, which formed triangular and extended linear clusters at higher concentrations, and exhibited sites of enhanced brightness around their edges. However, despite the fact that the samples used in all of these studies were from the same commercial source, the results are not entirely consistent. For instance, apparently similar defects were observed to be mobile at room temperature in one study (Namai et al., 2003), but immobile at temperatures up to 673 K in another (Esch et al., 2005), suggesting that the defects observed in the different experiments might not all be the same species, despite their similar appearances (Campbell and Peden, 2005).

A considerably greater number of studies have been performed on thin ceria(111) films grown on metallic substrates, including Rh(111) (Castellarin-Cudia et al., 2004; Chan and Yuhara, 2015), Ru(0001) (Lu et al., 2006; Zhou et al., 2008; Weststrate et al., 2009; Jerratsch et al., 2011; Hasegawa et al., 2014; Shahed et al., 2014), Cu(111) (Szabova et al., 2012; Hu et al., 2015), Pt(111) (Berner and Schierbaum, 2001; Grinter et al., 2010; Luches et al., 2011) and Au(111) (Zhao et al., 2007; Ma et al., 2008). The coverage, thickness and degree of structural order of the ceria films, as well as the resolution with which they were imaged, vary considerably, making direct comparison between them challenging. Depressions and protrusions, in isolation and in the form of clusters, are commonly observed, both in empty and filled state images.

In Table 1, we have attempted to summarize the experimental appearances and assignments of the features observed in filled and empty state STM images of CeO_2_(111), along with the experimental conditions (temperature and bias voltage), and substrate where applicable. We note that the results of different studies are not entirely consistent, with numerous different features being assigned to oxygen vacancies, with consideration of other possibilities being limited to an assignment of bright protrusions to hydroxyl groups in Shahed et al. (2014).

**Table 1:** Summary of experimentally observed STM features on ceria (111).

**Feature**	**Assignment**	**Bias (V)**	**T (K)**	**Sample**^**a**^	**Reference**
**Filled states**					
Depressions, extended lines	O vacancies	−2.5	773	Single crystal	Nörenberg and Briggs, 1997
Depressions, triangular	Triple O vacancies	−2.5	298	Single crystal	Nörenberg and Briggs, 1997
Protrusions, isolated and clusters	Not assigned	−2.5	298	Single crystal	Nörenberg and Briggs, 1997
Depressions, isolated and clusters	O vacancies	−3	298	Single crystal	Berner and Schierbaum, 2001
Depressions, isolated and clusters	O vacancies	−2	350	Single crystal	Namai et al., 2003
Depressions, isolated, triangular and lines	O vacancies	−3	573	Single crystal	Esch et al., 2005
Triple NNN Protrusions	Sub-surface O vacancies	−3	573	Single crystal	Esch et al., 2005
Depressions, isolated and clusters	O vacancies	−1	298	Au(111)	Zhao et al., 2007
Protrusions, isolated and clusters	Not assigned	−1	298	Au(111)	Zhao et al., 2007
Depressions/Protrusions, isolated and clusters	O vacancies	−1.5	298	Au(111)	Ma et al., 2008
Triple NNN Protrusions	Sub-surface O vacancies	−3	298	Pt(111)	Grinter et al., 2010
Depressions, isolated, triangular and lines	O vacancies	−3	298	Pt(111)	Grinter et al., 2010
Depressions, isolated	Not assigned	−4	573	Single crystal	Shahed et al., 2011
Protrusions, triangular	Hydroxyl groups	−4	150	Ru(0001)	Shahed et al., 2014
Depressions, triangular	O vacancies	−3	150	Ru(0001)	Shahed et al., 2014
**Empty states**					
Depressions, isolated and clusters	O vacancies	+0.5 to +1	298	Rh(111)	Castellarin-Cudia et al., 2004
Triple NNN Protrusions	Single O vacancy	+3	573	Single crystal	Esch et al., 2005
Large Y shaped clusters	Sub-surface O vacancies	+3	573	Single crystal	Esch et al., 2005
Depressions, triangular	Triple O vacancies	+2.5	298	Ru(0001)	Lu et al., 2006
Depressions, isolated and clusters	Not assigned	+3 to +4	298	Ru(0001)	Zhou et al., 2008
Depressions, extended clusters	Surface roughness	+3 to +4	298	Ru(0001)	Zhou et al., 2008
Depressions/Protrusions, isolated and clusters	O vacancies	+1.5	298	Au(111)	Ma et al., 2008
Depressions, isolated and clusters	Not assigned	+3	298	Ru(0001)	Weststrate et al., 2009
Depressions, clusters	O vacancies	+1 to +3	298	Ru(0001)	Zhou et al., 2010
Protrusions, double and triple	O vacancies	+1	10	Ru(0001)	Jerratsch et al., 2011
Depressions, clusters	O vacancies	+2.5	298	Ru(0001)	Zhou and Zhou, 2012

*Bias voltages have been rounded off to the nearest 0.5 V and room temperature is reported as 298 K*.

a *Ru(0001) means a ceria(111) film on a Ru(0001) substrate, for example*.

With the above discussion in mind, the principal purpose of the present paper is to offer a consistent set of simulated STM images of three particularly plausible candidate defects at anionic sites on CeO_2_(111), drawing attention to their similarities and differences, which we believe will aid in the interpretation of STM experiments on this system. We will also discuss other properties which could aid in distinguishing between them, making use of theoretical data available in the literature where available.

The three defects that we consider are oxygen vacancies, fluoride ions, and hydroxyl groups (denoted respectively OV, FI, and OH in the remainder of this paper). Our consideration of the OV defects is natural, given the well known ability and tendency of ceria to release oxygen under oxygen lean conditions, a quality which plays a central role in many of its technological applications (Trovarelli, 2013).

Surface OHs can arise due to the presence of H_2_O, which is a ubiquitous molecule even under ultra high vacuum conditions, and there are numerous reports that it reacts with OVs to form hydroxide ions embedded in the surface (Fronzi et al., 2009; Molinari et al., 2012; Mullins et al., 2012).

Finally, our consideration of fluoride ions is primarily due to the observation in Pieper et al. (2012) that fluorine was present in large quantities in single crystalline samples from the same source as those used in Nörenberg and Briggs (1997), Nörenberg and Briggs (1998), Nörenberg and Briggs (1999), Nörenberg (2002), Namai et al. (2003), Fukui et al. (2003), and Esch et al. (2005). Furthermore, it was observed in Zarraga-Colina et al. (2004) and Zarraga-Colina et al. (2005) that if CeO_2_ films are grown on CaF, F diffuses into the CeO_2_ upon annealing, replacing some of the O ions within the lattice. Taken together, these observations suggest that if CeO_2_ is exposed to a source of F, it is likely to become contaminated, and such contamination may be difficult to avoid entirely.

Previously, we concluded that, at least in filled state STM images, fluorine impurities and hydroxyl groups should be difficult to distinguish from oxygen vacancies at CeO_2_(111) based on their appearances alone (Kullgren et al., 2014), although OVs and FIs should be distinguishable based on their mobilities, relative concentrations in the surface and sub-surface layers, and tendency to form clusters in the surface layer. In this paper, we expand upon that study, providing a more detailed examination and comparison of the STM appearances of these three oxygen sub-lattice defects (OVs, FIs, and OHs), as well as discussing the results of related studies by other authors. We present both filled and empty state simulated STM images of the three defect species, both when located in the surface layer itself (section 3.2) and when located in the first oxygen sub-surface layer (section 3.3), and relate those images both to the local topography and to the electronic structure. In sections 3.4, 3.5 and 3.6 we discuss other distinguishing characteristics of the three defects, namely the positions of the f electron levels of the associated Ce^3+^ ions, the defects' mobilities and their distributions. We then conclude in section 4.

## 2. Computational Details

We carried out Kohn–Sham density functional theory (DFT) calculations using the projector augmented wave (PAW) formalism (Blöchl, 1994), as implemented in the Vienna Ab initio Simulation Package (Kresse and Hafner, 1993; Kresse and Furthmüller, 1996a,b; Kresse and Joubert, 1999) (VASP), version 5.3.5. We used the Perdew–Burke–Ernzerhof (PBE) functional (Perdew et al., 1996, 1997), augmented with a Hubbard-like (effective) *U* term in the simplified, rotationally invariant form introduced by Dudarev et al. (1998); Himmetoglu et al. (2014). The projectors that were used in the calculations of the occupation matrix were those associated with the PAW potentials, as is done by default in VASP, and we used a value of *U* = 5 eV (Nolan et al., 2005; Andersson et al., 2007; Castleton et al., 2007). The core electrons were described with the “standard” PBE derived PAW potentials distributed with VASP, in which the cores comprise the [Kr]4d electrons of Cerium and the 1s electrons of oxygen and fluorine. Aspherical contributions from the gradient corrections inside the PAW spheres were taken into account. The Kohn–Sham states for the valence electrons were expanded over the set of all plane waves of kinetic energy less than a cut-off value of 400 eV. Energies were converged to 1 × 10^−5^ eV, and forces to 1 × 10^−2^ eV/Å. We also used the Occupation Matrix Control (OMC) method in the implementation of B. Dorado and co-workers (Dorado et al., 2009) to avoid the problems associated with the existence of *electronic* self-consistent “local minima,” or “meta-stable" states, corresponding to different shapes and/or orientations of the *f* orbital that is occupied on a given Ce^3+^ ion.

For the structural model of CeO_2_(111), we used a periodic slab exposing a *p*(5 × 5) supercell of the (111) surface facet on both sides, with a thickness of three O–Ce–O triple layers; this leads to a formula for the stoichiometric slab of Ce_75_O_150_. A vacuum gap equivalent to the height of 5 bulk triple layers, which is more than 15 Å, was also used. Due to the large supercell used in our calculations, we found that it was sufficient to restrict sampling of the Brillouin zone to the Γ point.

Densities of states were produced by smearing the one-electron spectrum using a normalized Gaussian function with a width of 0.1 eV. Simulated STM images were produced under the Tersoff–Hamann approximation (Tersoff and Hamann, 1983, 1985), that is to say, directly from the local density of states (LDOS) obtained from partial charge densities calculated with VASP. The charge density was smeared with a Gaussian function with a width of 0.1 Å, and then linearly interpolated onto a fine 2D grid parallel to, and at a height 2.8 Å above, the upper surface of the slab; we found that increasing the distance any further introduced considerable numerical noise into the images. The partial charge densities were generated by summing the charge densities of all states within −3.0 eV or +3.0 eV of the Fermi level, assumed to be in the middle of the gap, for filled and empty state images, respectively. We note that the simulation protocol used here, which is based on the PBE+*U* functional, underestimates the band gaps somewhat, yielding O-2p → Ce-4f and O-2p → Ce-5d gaps of 2.25 and 5.20 eV, respectively. However, we find the simulated STM images to be rather insensitive to the precise value of the limit for the summation of states (and therefore the precise positions of the Fermi level and the band edges) with in general, although we present additional images when this is not the case.

## 3. Results

### 3.1. Preliminary Considerations

#### 3.1.1. Imaging the Stoichiometric Surface

**Figure 1:**
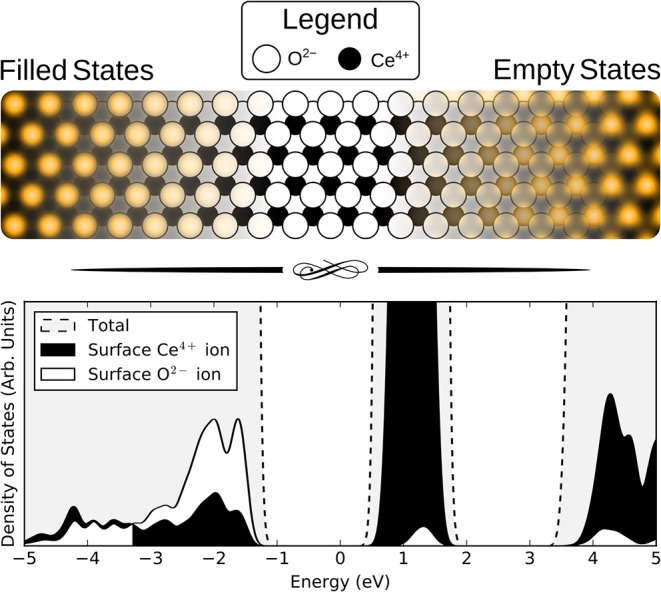
**(Upper)** Simulated STM images of the stoichiometric CeO_2_(111) surface using bias voltages of −3.0 V (filled states) and +3.0 V (empty states). The location of the ions is indicated by superimposing the STM images over a schematic top-view of the surface with white and black circles corresponding to oxygen and cerium ions, respectively. **(Lower)** Density of states (DOS) plots for the stoichiometric slab. Total DOS shown (gray) with dashed lines and projections onto a surface oxygen ion and a surface cerium ion are shown by white and black filled curves, respectively.

Simulated filled and empty state images of stoichiometric CeO_2_(111) are shown in Figure 1.

The (filled) valence band states of ceria derive (primarily) from 2p orbitals of the O^2−^ ions, with a smaller contribution from 4f orbitals of the Ce^4+^ ions in the layer beneath them, so it is natural that the topmost surface O^2−^ ions are what is imaged at negative bias voltages.

Regarding empty state images, some authors have suggested that they are due to electrons tunneling into conduction band states derived (primarily) from Ce 5d orbitals (Esch et al., 2005), rather than states derived (primarily) from Ce 4f orbitals, due to the more compact nature of the latter. However, the available experimental spectral data, summarized in Castleton et al. (2007), place the Ce 5d and 4f band edges at approximately 6 eV and 3 eV, respectively, above the valence band maximum (Wuilloud et al., 1984; Marabelli and Wachter, 1987; Pfau and Schierbaum, 1994; Mullins et al., 1998). It seems reasonable to assume that the Fermi level is below the unoccupied Ce 4f states, as it must be unless the sample is very heavily reduced indeed. We can therefore surmise that tunneling into the Ce 5d states is rather unlikely unless very high positive biases are used.

Given the above discussion, we therefore assume that in empty state imaging, electrons tunnel into states that derive mainly from Ce 4f orbitals. Note that the surface oxygen ions also contribute to the unoccupied states, and since they are in the layer above the Ce ions, they are also partly visible at positive bias voltages.

#### 3.1.2. Locations of Ce^3+^ Ions

All three defects introduce excess electrons into the lattice; in ceria, these electrons localize on individual Ce sites, reducing their charge states from +4 to +3, forming polarons. In order to maintain charge neutrality, OVs introduce two such Ce^3+^ ions, while FIs and OHs introduce one each.

The energetically preferential location of these Ce^3+^ ions in the vicinity of OVs has been the subject of much debate in the literature. The current consensus, based on theoretical calculations, is that the Ce^3+^ are located preferentially at next-nearest neighbor (NNN) sites to OVs (Murgida and Ganduglia-Pirovano, 2013; Sutton et al., 2015). In contrast, the single Ce^3+^ associated with an FI is preferentially located at a nearest neighbor (NN) site (Kullgren et al., 2014; Wolf et al., 2017), while for a surface OH, the two locations have essentially identical energies (Fernández-Torre et al., 2014; Kullgren et al., 2014; Wolf et al., 2017).

In principle, the Ce^3+^ ions (polarons) which charge compensate the substitional ions should break the threefold rotational symmetry of the lattice around the latter, but at higher temperatures, the electrons can hop between equivalent sites, so that the symmetry is restored (on average) on the time-scale required to obtain an STM image; indeed, this would appear to be the case for most of the experimental images in the literature, in which the rotational symmetry is preserved. For this reason, we present both images with the Ce^3+^ at specific locations, and images that have been rotationally averaged around the substitional ion.

### 3.2. STM Images of Defects in the Surface Layer

#### 3.2.1. Filled State Images

**Figure 2:**
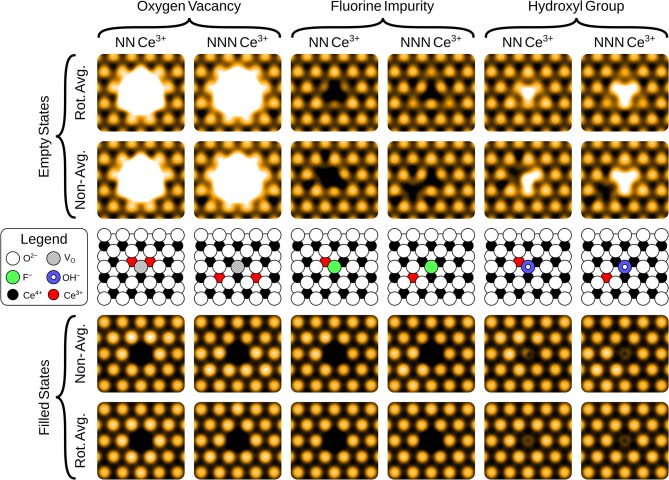
Simulated filled and empty state images of the three surface anionic defects, with the associated Ce^3+^ ions at either nearest neighbor (NN) or next nearest neighbor (NNN) lattice sites. The precise locations of the Ce^3+^ ions are shown in the schematics, for comparison with the non-rotationally averaged images.

The simulated filled state STM images of the three surface anionic defects, with the associated Ce^3+^ ions at either nearest neighbor (NN) or next nearest neighbor (NNN) locations, are shown in the bottom row of panels of Figure 2.

**Figure 3:**
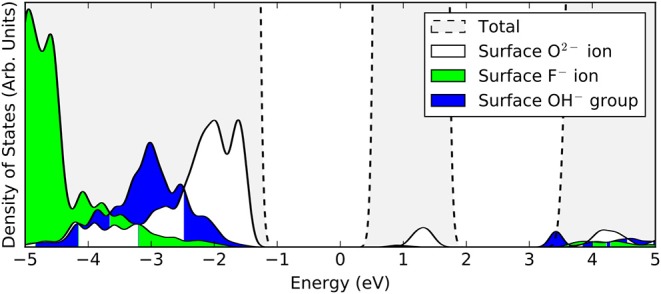
Projected densities of states (PDOS) of a surface oxygen ion (white), surface fluorine ion (green), and a surface hydroxyl group (blue). The total density of states of the stoichiometric slab (gray) is highlighted by the dashed lines.

In the filled state images, the main feature of all three defects is a dark “depression" at the lattice site of the defect center. For the surface OV, this is simply due to the absence of the ion and the associated electron density. The reason for the reduced tunneling current in the case of the fluoride or hydroxide ion in the filled state image can be understood by examining the partial density of states projected onto the defects (see Figure 3). Both the OH and the FI make their main contributions to the local density of occupied states at lower energies than the surrounding surface oxygen ions. Hence, despite the fact that both defects protrude from the surface, they are essentially invisible unless the bias voltage is large enough to allow tunneling from these states. At more negative bias voltages, both OH and FI begin to appear, with the OH being more visible than the FI for a given voltage, which in turn would be more visible than an OV. Thus, in principle, very sensitive STM measurements should be able to distinguish them by their relative apparent depth profile for a *fixed* bias voltage which probes states deep in the valence band.

Moving on to more subtle features of the STM images, we note that some of the oxygen ions in the vicinity of the central depressions are brighter than others. These features are correlated with the positions of the Ce^3+^ ions, and is easy to understand based on the fact that Ce^3+^ ions are larger than Ce^4+^ ions, and they are negatively charged with respect to the lattice. Both of these effects will cause the negatively charged O^2−^ ions to be repelled out of the surface. However, in some publications, an enhanced brightness was reported *between* certain pairs of the surrounding surface O ions (see e.g., Esch et al., 2005; Grinter et al., 2010). Such features have been explained as being due to a relaxation of pairs of surface O ions toward one another Esch et al. (2005). We found that we were only able to reproduce such effects using a *p*(2 × 2) surface supercell, as was done in Esch et al. (2005), which corresponds to a much higher defect concentration than that observed in the corresponding experiments. When we use larger *p*(4 × 4) or *p*(5 × 5) supercells, corresponding more closely to the experimental concentrations, we find a much weaker pairing, and furthermore it is rotated by 120°, i.e., it is between ions which are symmetrically inequivalent to those for which pairing was observed in the experiments in Esch et al. (2005). However, we note that our simulations are performed under the Tersoff–Hamann approximation (as were those in Esch et al., 2005), and thus (a) do not fully treat orbital directionality and (b) do not include the effects of the tip. Given the very low tunneling current of the measurements, it is possible that there are angular effects and/or non-negligible tip–surface interactions, which may be responsible for these features; in any case their origin remains unexplained. Furthermore, the pairing is not observed in all experiments (see Shahed et al., 2011 for example), which could be due to weaker tip–surface interactions.

#### 3.2.2. Empty State Images

**Figure 4:**
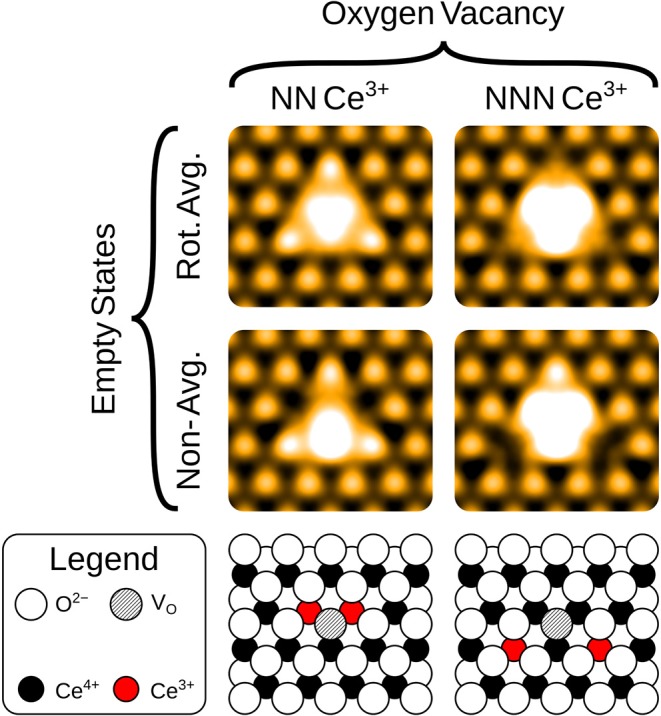
Simulated empty images at a bias voltage of 2.0 eV of a surface OV, with the associated Ce^3+^ ions at either nearest neighbor (NN) or next nearest neighbor (NNN) lattice sites. The precise locations of the Ce^3+^ ions are shown in the schematics, for comparison with the non-rotationally averaged images.

The empty state images of the three surface defects are given in the top row of Figure 2. At positive bias, the OV appears bright compared to its surroundings, due to a large, diffuse state centered on the vacancy, in agreement with images in Jerratsch et al. (2011). At +3.0 V bias the diffuse state spans the six closest cerium ions at the surface, while at a lower bias of +2.0 V, this diffuse state is transformed into a single or triple protrusion of Ce^4+^ ions neighboring the OV, as shown in Figure 4. The simulated empty state image of OH at positive bias voltages is also bright. Here, we find a rather localized feature at the OH, as compared to the more diffuse feature of the OV in the same bias voltage range. In contrast to the OV and OH, the FI is actually somewhat dark in the empty state image, which would make it more readily distinguishable from the other two defects than they would be from each other. In agreement with Jerratsch et al. (2011) we also find that Ce^3+^ ions appear dark due to their empty states being shifted toward higher energies.

### 3.3. STM Images of Defects in the Sub-Surface Layer

#### 3.3.1. Filled State Images

**Figure 5:**
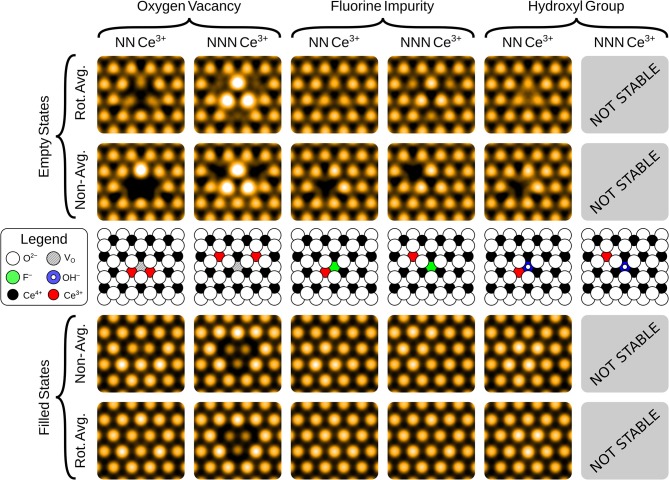
Simulated filled and empty state images of the three sub-surface anionic defects, with the associated Ce^3+^ ions at either nearest neighbor (NN) or next nearest neighbor (NNN) lattice sites. The precise locations of the Ce^3+^ ions are shown in the schematics, for comparison with the non-rotationally averaged images.

The filled state STM appearances of the defects in the first oxygen sub-surface (third atomic) layer are shown in the bottom row of Figure 5. In contrast to the surface defects, their appearance is due primarily to the effect the defects have on the surface layer ions above them, rather than imaging the defect site itself. Hence, the apparent topography is a closer reflection of the actual surface topography (i.e., the relative heights of the surface ions).

Triangular protrusions in experimental filled state images have been ascribed to sub-surface oxygen vacancies (Esch et al., 2005; Grinter et al., 2010; Jerratsch et al., 2011). Our simulated images of a sub-surface OV do produce such a protrusion, although we also find that it should be accompanied by a significant depression of the three closest surface oxygen ions, which relax into the surface due to the presence of the vacancy beneath them. The magnitude of this effect depends upon the locations of the Ce^3+^ ions; in the lowest energy NNN configuration, the ions relax downwards by 0.19 Å, while with NN localization, this is reduced to 0.09 Å.

For the sub-surface FI, the main effect for NN localization is the outward relaxation by 0.05 Å. This is also seen if the electrons are at NNN positions, but then it is accompanied by a slight inward relaxation of the three nearest surface O ions, by 0.04 Å. Both of these effects are similar for the OV, but more subtle due to the smaller degree of relaxation.

The sub-surface OH is somewhat different from the other two defects. The triple protrusion is again present, and the associated displacement of the ions is of similar magnitude to the surface FI with NN localization, namely 0.05 Å. However, the more striking feature is the apparent protrusion of the three nearest neighbor oxygen ions, which are pushed out of the surface by the OH by 0.05 Å. This produces a small, bright triangle with the opposite orientation relative to the underlying lattice, as compared to the sub-surface oxygen vacancy. A careful analysis of the partial density of states suggests that the reason why these ions appear brighter, despite being at a similar height, is that the states associated with them are pushed up slightly in energy, likely due to the presence of the proton between them.

#### 3.3.2. Empty State Images

The empty state images, shown in the top row of Figure 5, all follow similar patterns. Again, the Ce^3+^ ions appear as dark depressions in all cases, due to the unoccupied states being pushed upwards in energy. For the OV, with NNN localization, we also see a triangular protrusion associated with the Ce^4+^ ions being repelled upward by the defect. We note that this pattern was suggested to be the fingerprint of a surface OV in the combined experimental and theoretical work of Jerratsch et al. (2011). While their observations in empty state images were matched to a single protrusion at negative bias, identification based solely on empty state imaging could lead to mis-interpreting sub-surface OVs as surface OVs, especially at low positive bias where the two appear very much alike (see Figure 4). There is a similar, but more subtle protrusion for the FI.

### 3.4. f-Electron Spectral Features

**Figure 6:**
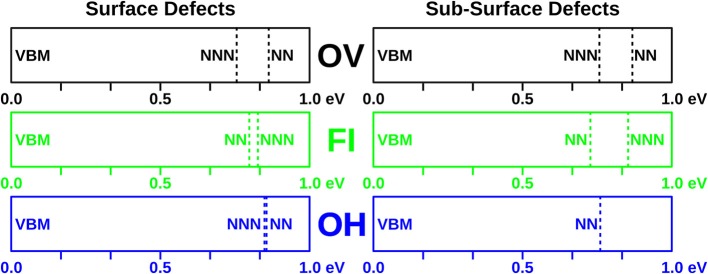
f electron eigenvalues of the defects studied in this work. Positions of f-levels with respect to the valence band for all defects with nearest (NN) and next nearest neighbor (NNN) localization are indicated.

Scanning tunneling spectroscopy (STS) has been performed on CeO_2_(111) thin films to determine the energy levels of localized f electrons on Ce^3+^ ions located near defects. This provides another possible way to distinguish between defects that share the same gross appearance. Therefore, we have plotted in Figure 6 the eigenvalues of the f-electron states associated with all of the defects studied herein. They fall in a range from 0.72 to 0.86 eV with respect to the valence band edge. In the combined experimental and theoretical work by Jerratsch et al. (2011) such spectral features were used to discriminate between defects with different Ce^3+^ localization patterns.

### 3.5. Diffusion Rates

The diffusion barrier for OVs at CeO_2_(111) has been calculated by a number of authors (Plata et al., 2013; Su et al., 2016). The values are generally quite small, i.e., below 0.5 eV, which is consistent with the experimentally observed high ionic conductivity of the material; indeed, this is a property of the material which is exploited in many of its technological applications.

In Kullgren et al. (2014), a diffusion mechanism of the FI was considered which involves it moving out of the lattice position to a site on top of a neighboring Ce^4+^ ion; with the OV thus formed moving simultaneously to a sub-surface position. The barrier height was calculated to be 1.47 eV, which we re-calculate using our current computational set-up to be 1.42 eV.

In Fernández-Torre et al. (2014), a diffusion barrier was presented for surface OH groups. The mechanism involves the transfer of the proton between a surface and sub-surface oxygen site, with a reported activation energy of 1.8 eV. Here, we considered an alternative mechanism similar to that of FI diffusion, in which the entire OH group moves up on to a surface Ce^4+^ ion. We find the barrier height for such a process to be 1.50 eV, i.e., 0.3 eV lower than the barrier reported in Fernández-Torre et al. (2014), and much more similar to the barrier for FI diffusion.

We used these values to calculate diffusion rates from an Arrhenius expression at 77 K, 300 K and 600 K, along with a prefactor of 1 × 10^13^_s^−1^_. These values, are collected in Table 2. Furthermore, given that the diffusion path for the latter two defects considered here requires the concerted motion of both the defects themselves, and a sub-surface oxygen ion, the actual rate is likely to be lower than that calculated here. We therefore conclude that OVs are too mobile to be imaged by STM, for which a single scan takes on the order of minutes, unless experiments are conducted at temperatures significantly lower than room temperature. FIs and OHs on the other hand, should be immobile for sufficiently long for them to be imaged within the full range of temperatures used in experiments reported in the literature.

**Table 2:** Defect diffusion rates of isolated defects (in s^−1^) from the Arrhenius equation, using a pre-factor of 1 × 10^13^_s^−1^_.

**T (K)**	**OV**	**FI**	**OH**
77	1.88 × 10^−20^	1.47 × 10^−80^	7.60 × 10^−86^
300	3.98 × 10^4^	1.49 × 10^−11^	6.55 × 10^−13^
600	6.31 × 10^8^	1.22 × 10^1^	2.56

We note that it has been demonstrated that migration of charged defects on the ceria surface could proceed via a diabatic channel, in which case the activation energy barriers may be drastically increased, by as much as a factor of three (Lustemberg et al., 2016). However, this only applies to migrating species that change their charge state during the transition. For the defects considered here the migrating species remain in the same charge state in the initial, final and transition states.

### 3.6. Defect Distributions and Interactions

For non-interacting defects of a given species, the proportion of surface to sub-surface defects at thermal equilibrium can be determined by evaluating an appropriate Boltzmann factor. The energy difference between an isolated surface and sub-surface defect of the same type is +0.12 eV for the OV, −0.48 eV for an FI and −1.45 eV for an OH (with a negative value indicating greater stability in the surface layer), according to our calculations. Using these values to calculate the Boltzmann factors at 600 K (within the range of temperatures used in Nörenberg and Briggs, 1999; Nörenberg, 2002; Namai et al., 2003; Esch et al., 2005) suggests that the number of surface OVs for each sub-surface OV is at most 1 × 10^−1^, for FIs at least 1 × 10^4^ and at least 1 × 10^12^ for OHs. These values, along with analogous ones calculated for *T* = 77 K and *T* = 300 K, are collected in Table 3, and can be summarized by saying that under most conditions one would expect to see OVs in the sub-surface layer, but FIs and OHs in the surface layer.

**Table 3:** Relative proportions of isolated surface to sub-surface defects of a given species from Boltzmann factors.

**T (K)**	**OV**	**FI**	**OH**
77	1.40 × 10^−8^	2.61 × 10^31^	8.03 × 10^94^
300	9.64 × 10^−3^	1.16 × 10^8^	2.29 × 10^24^
600	9.82 × 10^−2^	1.08 × 10^4^	1.51 × 10^12^

However, at higher defect concentrations, interactions between defects will necessarily become important; indeed, one of the enduring points of interest regarding the defects observed in experimental images is their distribution, and in particular, their tendency to form linear and triangular clusters at the surface. DFT simulations have shown that surface OVs do not exhibit any tendency toward NN clustering (Conesa, 2009; Zhang et al., 2009; Sutton et al., 2015), based on comparisons of total energies. On the other hand, both surface FIs (Kullgren et al., 2014) and OHs have been calculated to be stable as constituents of NN dimers (Fernández-Torre et al., 2014) although the binding energies are small (< 0.1 eV/dimer).

Furthermore, combinations of surface OVs, along with sub-surface OVs or sub-surface OHs have also been considered in the DFT study in Wu and Gong (2016), although all of those combinations were shown subsequently to be unstable with respect to decomposition into their isolated constituents (Wolf et al., 2016).

The above conclusions are based solely on differences in total energies, but more recently, the importance of configurational entropy in determining distributions of OVs and FIs has been studied. In Kullgren et al. (2017), DFT-based Monte Carlo simulations showed that OHs have far less tendency to form NN clusters than FIs, and that OHs instead tend to form clusters in which they are located at next-nearest-neighbor sites. For FIs, large and compact clusters are most abundant at 300 K while at 600 K straight and hooked linear clusters are abundant.

DFT based Monte Carlo calculations have also been performed including both surface and sub-surface OVs at a variety of temperatures and levels of reduction (Han et al., 2018). At lower concentrations (10% and 15%) and/or temperature (80 K) the number of NN clusters at the surface was found to be very small, although the numerous sub-surface vacancies do exhibit p(2 × 2) ordering, consistent with the interpretation of low temperature AFM experiments (Torbrügge et al., 2007). Meanwhile at higher temperatures and levels of reduction, numerous linear NN clusters were observed.

However, standard Monte Carlo simulations such as those described above do not take into account kinetic effects, which must be significant due to the high mobility of OVs. Indeed, the surface OV clusters are reported by Han et al. to be abundant only in simulations performed at temperatures at which OVs are expected to be mobile on the time-scale of STM experiments (see Table 2). Although it is conceivable that OVs which comprise parts of clusters could be significantly less mobile than isolated OVs, calculations that address this possibility have not been reported.

## 4. Conclusion

STM images of the ceria (111) surface exhibit a number of point defects in an otherwise regular hexagonal pattern. These features are commonly assigned to surface oxygen vacancies, although alternative interpretations have been proposed. In this contribution, we have used Density Functional Theory (DFT) with the PBE+*U* functional to examine three likely candidates for anionic defects on CeO_2_(111), namely oxygen vacancies (OV), fluorine impurities (FI) and hydroxyl groups (OH), in both the surface and the first sub-surface layers. We have presented and analyzed a consistent set of simulated STM images for them under the Tersoff–Hamann approximation.

Based on our results we suggest that missing spots in the *filled state* images at the surface oxygen lattice could be caused by *any* of the three defects considered here. However, the corresponding empty state images of the three defects are rather different to one another. The clean surface in this case shows spots at Ce sites, and against this background, surface OVs appear as large, bright features centered at oxygen sites, and OHs give smaller but more intense features also at oxygen sites. The FI signature is also centered on an oxygen site, but consists of a triangle of darkened Ce ions surrounding it, relative to the background lattice.

The appearance of the sub-surface defects in filled state images is mainly a reflection of topographical changes at the surface (relaxation of ions) induced by those defects. In filled state images, OVs produce triangular protrusions of next nearest neighbor surface oxygen ions similar to those observed in some experiments (cf. Esch et al., 2005; Grinter et al., 2010), but they also produce a rather striking triangular depression *inside* the protruding triangle, to an extent that may in fact render it difficult to distinguish from a triangular surface defect cluster of either OV, OH or FI. The FI reproduces both of these features, but the contrast variations are more subtle, while the OH produces only protrusions. In the empty state images we note that Ce^4+^ which are nearest neighbors to a sub-surface OV appear bright. A similar but much more subtle effect is also seen for sub-surface FI.

In general, our simulations suggest that missing spots in the *empty state* images, at the cerium lattice, can be caused by Ce^3+^ that are *not* nearest or next nearest neighbor to a surface vacancy, or nearest neighbor to a surface hydroxide.

Regarding their physical properties other than appearances, calculations indicate that OVs are most stable at the sub-surface and that they diffuse rapidly unless the temperature is kept much below room temperature. OVs do not have a strong *energetic* tendency to cluster, although DFT-based Monte Carlo simulations suggest that, given sufficient vacancy concentration and temperature, clusters can emerge. However, their amounts seem to be appreciable only when the temperature is too high for them to be imaged. In contrast, both FI and OH are quite stable at the surface and diffuse slowly. FI's show a tendency for clustering in the surface layer over a wide range of temperatures and concentrations, with OH representing an intermediate case.

## Author Contributions

MJW and JK performed the calculations and prepared the first version of the manuscript. All authors were involved in the planning of the work, the analysis and interpretation of the results and in the finalization of the text.

### Conflict of Interest Statement

The authors declare that the research was conducted in the absence of any commercial or financial relationships that could be construed as a potential conflict of interest.
